# A Glimmer of Hope: Maintain Mitochondrial Homeostasis to Mitigate Alzheimer’s Disease

**DOI:** 10.14336/AD.2020.0105

**Published:** 2020-10-01

**Authors:** Wenbo Li, Ling Kui, Tsirukis Demetrios, Xun Gong, Min Tang

**Affiliations:** ^1^State Key Laboratory of Natural and Biomimetic Drugs, School of Pharmaceutical Sciences, Peking University, China.; ^2^Dana-Farber Cancer Institute, Harvard Medical School, United States; ^3^Boston Children’s Hospital, Harvard Medical School, United States; ^4^Department of Rheumatology & Immunology, The First Affiliated Hospital of Anhui Medical University, China; ^5^Institute of Life Sciences, Jiangsu University, China; ^6^Center for Innovation in Brain Science, University of Arizona, United States

**Keywords:** Alzheimer’s disease, mitochondria dysfunction, mitophagy, NAD+

## Abstract

Mitochondria are classically known to be cellular energy producers. Given the high-energy demanding nature of neurons in the brain, it is essential that the mitochondrial pool remains healthy and provides a continuous and efficient supply of energy. However, mitochondrial dysfunction is inevitable in aging and neurodegenerative diseases. In Alzheimer’s disease (AD), neurons experience unbalanced homeostasis like damaged mitochondrial biogenesis and defective mitophagy, with the latter promoting the disease-defining amyloid β (Aβ) and p-Tau pathologies impaired mitophagy contributes to inflammation and the aggregation of Aβ and p-Tau-containing neurotoxic proteins. Interventions that restore defective mitophagy may, therefore, alleviate AD symptoms, pointing out the possibility of a novel therapy. This review aims to illustrate mitochondrial biology with a focus on mitophagy and propose strategies to treat AD while maintaining mitochondrial homeostasis.

## 1. Introduction

Alzheimer’s disease (AD) is the most frequent cause of dementia, affecting around 50 million individuals in 2018. By 2050, it will rise to 152 million [1]. AD prevalence is low among persons younger than 65 years, but increases to 10% to 30% among persons older than 85 [2-4]. Neuropathologically, the disease-defining histo-pathological abnormalities, including extracellular deposits of amyloid-β (Aβ) peptide and intraneuronal accumulation of hyperphosphorylated tau (p-Tau), spread through the brain in a nonrandom manner with early pathology occurring in the entorhinal cortex and hippocampus [5-8]. Efforts made in AD research during the last several decades have provided essential insights into the pathogenesis of AD, but the molecular mechanisms are not fully understanding recovered. Numerous standard and rare susceptibility AD-associated genes had found. Which can confer small etiologic origination [9-12]? Although AD was first discovered in the late 19th century[13], an effective treatment has yet to develop. Moreover, there has not, however, been a pharmacological method created to fundamentally cure AD, with mounting Anti-AD drugs ‘dying’ in phase III clinical trials [14].

Excluding the aggregate of Aβ peptides extracellularly and microtubule-associated p-Tau intracellularly [15, 16], the AD research community increasingly believes that the accumulative damaged mitochondrial biogenesis in the brain mainly results from deficient mitochondrial autophagy (mitophagy), contributing a lot to AD pathogenesis [17, 18]. This review aims to discuss both the pivotal mammalian components of mitochondrial biology and aspects of mitophagy and the possible strategies to maintain mitochondrial homeostasis.

## 2. Mitochondrial integrity and regulation

### 2.1 Mitochondrion in reasonable condition

**Figure 1.**
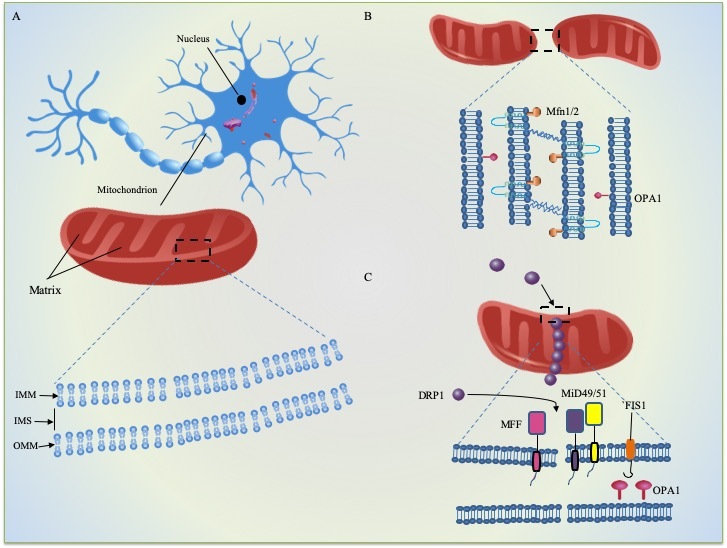
Mitochondrial cell biology. (A) structure of mitochondrion and general features of mitochondrial dynamics (B) fusion (C) fission.

Mitochondria vary tremendously across cell types and tissues in morphology and allow for rapid changes in response to external lesions and metabolic prompts. Generally, there are multiple copies of mitochondria within cells, with diameters ranging from 0.75 and 3um. However, all mitochondria are well known as the energy factories within various types of cells, employing the oxidative phosphorylation (OXPHOS) process to produce Adenosine triphosphate (ATP). The mitochondrion in most eukaryotic cells is a double-membrane-bound organelle comprised of a phospholipid bilayer. As Figure 1A shows, it has an inner mitochondrial membrane (IMM) with numerous folded cristae, a smooth outer mitochondrial membrane (OMM) and the intermembrane space (IMS) between them. The mitochondrial morphology is dramatically shaped by ongoing fusion and fission (see Fig. 1B and 1C) on IMM and OMM [19, 20]. Beyond the IMM is the jelly-like matrix for respiration, where the breakdown of pyruvate into adenine triphosphate (ATP) and the tricarboxylic acid (TCA) cycle takes place [21, 22]. Additionally, the electron transport chain (ETC) locates at the IMM, which is essential for the generation of ATP via oxidative phosphorylation machinery [23].

In addition to its most essential functions of energy production, ROS production, and Ca^2+^homeostasis, mitochondria also play a role in apoptosis [23]. Cell apoptosis can trigger by the release of cell death signaling molecules from the mitochondria to the cytoplasm through the permeabilization of OMM [24]. This mitochondrion-involved apoptosis modulated by three groups of Bcl-2 family proteins: Bcl-2-like proteins, Bax/Bak proteins, and BH3 proteins [25]. The OMM permeabilization is caused by the channel within the OMM formed by Bax/Bak proteins. Through this channel, the caspase protein, a key signaling molecule, is released into the cytoplasm. After activation by cytochrome C,Smac and Omi, the apoptosome is formed and activated, causing severe cell death [25].

### 2.2 Mitochondrial biogenesis, degradation, and their regulations

Mitochondrial productions show an interaction present between the nuclear and mitochondrial genomes. In the mammalian genome, 37 genes have been defined in mitochondrial DNA (mtDNA. Of these genes, 13 are mRNAs that encode polypeptides for OXPHOS, and the rest aid in the transcription and translation within the organelle, with 22 being tRNAs and 2 being rRNAs [26-31]. However, the nuclear genome encoded around 1500 kinds of proteins and translated on cytoplasmic ribosomes, where they are eventually imported into mitochondria by the translocase of the inner membrane (TIM) and the outer membrane (TOM) [32]. This dual origin implies that coordinate mitochondria. Phospholipids are either directly synthesized in the organelle or imported from the endoplasmic reticulum membrane after synthesis, along with many cofactors and metals. The OMM has a similar composition to the rest of the cell and contains a pore formed by the β-barrel protein voltage-dependent anion channel (VDAC) through which interchange happens between the cytosol and the IMS [32]. The IMM greatly enhances its area by infolding into cristae in the shape of gracile sacs and contains a large amount of the phospholipid cardiolipin on the surface. In a sense, the crista is a supporting measure for very useful OXPHOS [19] and has the vast majority proteases. As the first phase of mitochondrial quality control, a mitochondrion can clean low levels of damaged proteins with the aid of intraorganellar proteases and chaperones, for instance, HTRA2 and TRAP1 [33].

Mitochondria continually divide, blend, and alter their size, knitting a dynamic network within the cell, and their movement and turnover in the matrix coordinated by the cytoskeleton [34]. These processes collectively termed as mitochondrial dynamics. In other words, to maintain both of the quality and quantity or meet the needs of cells/tissues, mitochondria go through constant fusion and fission. The latter recognize as one core mitochondrial quality-control pathway [33]. Balanced fusion and fission can shape the final mitochondria to fit mitochondrial metabolism and ensure the removal of other dysfunctional organelles [20]. Mitochondrial degradation happens in response to cellular damage and nutrient excess and has documented in cancer, obesity, and cardiovascular and neuromuscular disorders [26, 28, 35-39]. The autophagic clearance of mitochondria facilitated to adapt the mitochondrial activities to satisfy the physiological needs[40].

## 3. Mitophagy- mitochondrial autophagy

### 3.1 Autophagy and autophagy flux

Macroautophagy, commonly known as autophagy, is a major intracytoplasmic pathway for the elimination of damaged organelles and protein aggregates, recycles cellular biomaterial. It is a conserved lysosomal degradative process whereby obsolete cellular constituents are delivered by double-membrane vesicles (called autophagosomes) to the lysosome for degradation. The dynamic process of autophagy presented with the term ‘autophagic flux.’ It mainly includes autophagosome formation, maturation, fusion with lysosomes, and subsequent decomposition and release of the remaining molecules into the cytosol [41, 42]. In AD, Aβ, one of its pathological hallmarks, may be degraded by autophagy or even be reduced by the upregulation of autophagy in many in vivo and in vitro systems [43-46]. Additionally, aberrant Tau induces an increase in the number of autophagosomes and contributes to toxicity in AD [47, 48]. The in vitro experiments and the mouse model showed that Tau binds lysosomal membranes to change its permeability [49, 50]. Perez *et al*. indicated that defective lysosomal membrane integrity contributes to AD onset, which is independent of Tau or Aβ pathology [51]. In a word, several studies demonstrated that autophagic flux defects are closely correlated to the pathogenesis of AD [52-54], and the overall investigations predict that modulations of autophagy are therapeutic opportunities for AD [55, 56]. However, the autophagy pathway is involved with multiple steps and different modes of regulation which makes it complicated.

### 3.2 Mitophagy cell biology and detection

Compared with nuclear DNA, disruption of mitochondrial dynamics results in reasonably high ROS, which causes more oxidative damage to mtDNA as there is no protection of associated histones and other chromatin proteins. These damaged mitochondria are selectively marked, elongated by phagophores to form a mature mitophagosome, and fused with lysosomes where they degrade as autolysosomes. This macroautophagy process, namely mitophagy, is one of the mitochondrial quality control pathways used to guarantee basal mitochondrial turnover [57-59]. In addition to the ROS inducer, mitophagy also can be induced by some physiological conditions, including the maturation of erythrocytes and the development of fertilized oocytes [60, 61]mitochondrial dynamics and mitophagy, cells can have stringent quality control mechanisms to maintain a healthy mitochondrial population [62].

All types of mitophagy are mainly modulated by the (PTEN-induced putative kinase 1) Pink1-Parkin pathway, whose primary target is the mitochondrion devoid of the membrane potential (ΔΨm) [63]. In normal circumstances, Pink1 is predominately expressed on the IMM, translocated to the cytosol, and digested by the proteasome. However, when mitochondria lose biological functions, the Pink-Parkin pathway is immediately activated. Instead of being degraded by the proteasome, Pink translocated to the OMM, which results in its accumulation on the OMM [64-66]. Subsequently, Pink stabilizes on OMM and promotes the recruitment of Parkin, whose E3 ligase activity launched. Parkin then ubiquitinates several components on the OMM to format poly-ubiquitin chains. After subsequent phosphorylation by Pink1, the poly-ubiquitin chains release ‘clean me’ signals for autophagic machinery. As a result, dysfunctional mitochondria are recognized and bound by adaptor proteins (such as p62, OPTN, NDP52) through the poly-ubiquitin chains followed by the elongation, maturation, and enclosure of the double membraned phagophore [67]. The autophagosome formation is initiated by binding with LC3, and finally, the matured autophagosome fuses with the acidic lysosome for the degradation and recycling of the engulfed mitochondria. Moreover, independent of Parkin, autophagosomes can be recruited to mitochondria via direct interaction with LC3 by increasing the expression of FUNDC1 and NIX in mammalian cells [68, 69]. Some ubiquitin ligases can target depolarized mitochondria for mitophagy, such as SMURF1 [70, 71]. Other molecular mechanisms of mitophagy have been extensively studied [16, 72, 73]. Collectively, the major mitophagy pathways include the PHB2 pathway, the cardiolipin pathway, the NBR1 pathway, the Tax1 binding protein 1 (TAXIBP1) pathway, the OPTN pathway, the BNIP3 pathway, the BCL2L13 pathway, the FKBP8 pathway, the AMBRA1 pathway, and the NDP52 pathway. A detailed description of these pathways has summarized in recent reviews [72-74]. Remarkably, Lu *et al*. found a new class of adaptors, namely CUET proteins, can bind ubiquitylated substrates in yeast, implying the potential variety of the complements in the above pathways [75]. Emerging evidence suggests that mitochondrial dynamics affect the homeostasis of mitophagy. As mitochondrial fusion and fission have implicated in many of the classical mitochondrion-associated cellular pathways such as calcium signaling, apoptosis, and the cell cycle, it generally believes that mitochondrial fission facilitates mitophagy as smaller mitochondria are easier for autophagic engulfment [34, 76]. Thus, it is critical to correctly measuring mitophagy. There are several versatile methods to quantify mitophagy in human cells, *C. elegans* (e.g., Rosella and DCT-1/LGG-1 strains), flies (e.g., mito-QC and mito-Keima strains). Besides, the mito-QC and mito-Keima stains in mice are also available, enabling a temporospatial evaluation of mitophagy [73, 77-81].

Interestingly, Paasch *et al*. proposed that nonfunctional mitochondrial proteins modestly induced by failed mitochondrial import can mark by SUMO (small ubiquitin-like modifier). Therefore, SUMOylation can serve as intraorganellar protein quality control [82]. Since mitophagy is a constant, dynamic and sophisticated cellular process, a combination of different mitophagy detection approaches, including cross-species evaluation, will improve the accuracy of measuring mitophagy and lead to a better understanding of mitophagy in the physiology of neurons, potentially contributing valuable information to the effort of advancing the therapy of neurodegenerative diseases.

### 3.3. Compromised mitophagy in AD

Although the exact mechanism is not precise, emerging findings suggest that impaired mitophagy contributes to neuronal dysfunction and cognitive decline by trigging Aβ and p-Tau accumulation in AD pathophysiology [16, 28, 83]. These protein aggregations can produce adverse effects on mitochondrial functions, such as a disruption to the membrane potential resulting in less ATP production. This disruption also can change the permeabilization of the OMM by opening mitochondrial permeability transition pore (mPTP) [23]. Moreover, intra-mitochondrial Aβ has been demonstrated to interplay with ABAD (Aβ binding alcohol dehydrogenase) than to generate ROS [33]. Partly due to these processes, the neurons in AD patients experience mitochondrial dysfunction, which causes a bioenergetic deficit early and promotes the Aβ and p-Tau pathologies [16]. Mitophagy predominantly regulates mitochondrial dynamics and the timely clearance of dysfunctional mitochondria that is necessary for the maintenance of synaptic plasticity, neuronal function and neuronal survival. Mitophagy in neurons is essential to prevent neuronal death and pathogenic brain aging via targeting the superfluous or dysfunctional mitochondria by lysosomes [72, 84, 85]. Cumulative evidence reveals that compromised mitophagy contributes to aging and neurodegeneration observed in models of premature aging disease and AD [16, 77, 85-88]. Mitophagy deficits and accumulation of mitochondria detected in AD human post-mortem tissue as well as reduced phosphorylation of mitophagy initiation proteins (ULK1, TBK1) and elevated levels of mitochondrial membrane proteins (COXIV, TOMM20) [77, 88]. Increasing experiments from *C. elegans*, murine and human cell lines overexpressing WT and mutant tau implicate impaired mitophagy, which leads to the accumulation of dysfunctional mitochondria and the impairment of cognitive deficits [77, 86, 88].

Impaired autophagic flux related to mitochondria is another contributor to the neurodegeneration of AD. The defects may arise from any of the critical processes mentioned above. Unfortunately, the mechanisms underlying the deficiencies in AD are not fundamentally understood. As mentioned earlier in this review, autophagic flux consistently and efficiently initiated by autophagosomes, also called autophagic vacuoles (AVs) followed by formation, maturation, fusion, and digestion within lysosomes. Induced autophagy causes the AV aggregations in AD, impairment of digestion/breakdown steps, and a high rate of autophagosome formation companying with inefficient fusion with lysosomes [89]. Boland *et al*. proved that blocking the clearance of autophagosomes in cultured neurons by restraining lysosomal proteolysis results in rapid and marked AV accumulation [43]. Nevertheless, the autophagosomes accumulation in the AD brain does not mean that autophagy initiation is upregulated [90, 91].

On the contrary, one of the essential initiators, Beclin-1 cleaved by caspase three enzyme is low expressed in AD patients [92]. In an APP transgenic mouse model, the deletion of Beclin-1 stops autophagy and accelerates Aβ accumulation [90]. In addition to the defects in the initiation process, genetic or functional alterations may exist in autophagosome maturation, formation, or clearance processes [89, 93, 94]. Taking the mutations in presenilin-1 as an example, the primary defect in lysosome acidification and proteolysis causes pathogenic protein accumulation in AD.

**Figure 2.**
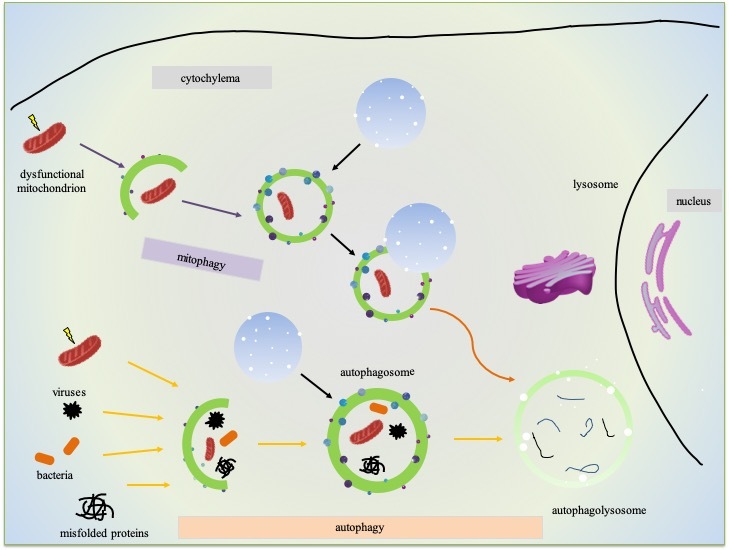
Overview of autophagy/mitophagy in neurons.

The neurodegeneration of AD directly linked to autophagic flux, and there are several methods developed to identify the status of autophagic fix [42]. The most straightforward approach in experiments for monitoring autophagy is Atg8/LC3 detection and quantification [95, 96]. Autophagic flux perceives by LC3-II turnover with the aid of western blot in the absence and presence of lysosomal degradation inhibitors, such as E64d, pepstatin A, NH_4_Cl, bafilomycin A1 and chloroquine. The signal of LC3-II increases in the presence of one of the above inhibitors when the autophagic flux is occurring as the transition of LC3-II is blocked [97-99]. The other two methods are assays for degradation of SQSTM1/p62 and long-lived proteins, for details, please refer to [42, 100]. (Fig. 2)

### 3.4 Impaired proteostasis related to mitophagy

The phenomena of protein misfolding and aggregation presented in many kinds of neurodegenerative disease studies. Presently, each of them characterized neurodegenerative diseases with at least one pivotal protein misfolded, which serves as a clinical biomarker. Usually, this protein repeatedly generated and aggregated, along with the disease exacerbation [101]. In AD, the most significant manifestation of misfolded proteins is cerebral plaques. P-Tau proteins and Aβ peptides separately form neurofibrillary tangles (NFTs) and Aβ plaques. Reasonably, the cellular proteostasis in AD-related to mitophagy is easily impaired, including those involved in mitochondrial biogenesis (PGC-1α) [102], mitochondrial responses to oxidative [103] and bioenergetic challenges (SIRT3) [104], mitochondrial fission and fusion (Drp1) [105]. Lysosomes are the protease pool in cells that play the role of disintegrating the ultimately damaged and dysfunctional molecules. Nevertheless, the experiments on cell culture and AD animal models illustrated that mitochondrial concomitants emerged from the excessive fission as a result of lysosomal dysfunction [106, 107].

**Table 1:** Summary of different pathways and genes/proteins involved in autophagy/mitophagy.

Pathways	Genes/Proteins	Refs
The PINK1/Parkin-dependent pathway	PINK1, Parkin, USP8, USP30, AMBRA1, Bcl2, FUNDC1, MUL1, Nix, ATG5, LC3	[74, 119]
The Parkin-independent pathway	PINK1	[120]
The Reticulocyte pathway	Nix, LC3	[60]
The Zygote pathway	VCPs, MUL1, LC3	[121-123]
The MDVs pathway	PINK1, Parkin, LC3	[124]
The mTOR pathway	ULK1, Atg1, Atg13, Akt, PKC, FOXO3	[45]

In mammals, about 20 proteins are involved in mitophagy, including Drp1, mitofusin, PGC-1α, SIRT, ULK1, BNIP3L/NIX, TBK1, PINK1, and Parkin (see Table 1). Several studies have declared that increased levels of Drp1 in the brains of AD mice and patients were detected while some others have conflicting results [108, 109]. Analyses of postmortem brain tissues of AD patients and age-matched controls have uncovered reduced expression of genes associated with mitochondrial biogenesis, including TFAM, PGC-1α, and NRF2. The mitochondrial SIRT3 and nuclear SIRT1 are NAD^+^-dependent deacetylases, which may inhibit AD through the modulation of many cellular pathways. The reduction of these two proteins may link to neurodegenerative diseases. Studies in the parietal cortex of AD patients shown that reduced SIRT1 expression is closely associated with the accumulation of Tau tangles and Aβ plaques [110]. Notably, SIRT1 can upregulate PGC-1α and participate in the mitophagy induction through the activation and deacetylation of the major autophagy proteins (ATG8/LC3, ATG5, ATG7) and the upregulation of the mitophagy proteins (NIX/BNIP3L, LC3) [85, 87]. Martine and her colleagues provided a mechanism for cell death with the axis of PAPR-1_NAD^+^_SIRT1_PGC-1α, which links nuclear DNA damage to mitochondrial homeostasis. They explained that activated PARP-1 (the enzyme poly(ADP-ribose) polymerase1) contributes to AD by the accumulation of ADP-ribose polymers and energy crisis [84, 111]. A steep reduction of NAD^+^ levels can induce through the increase of PARP-1 activity, with the consequence of mitochondrial impairment [112]. Genetically, mutations in genes encoding the proteins outlined above contribute to the etiology of AD [113, 114]. Furthermore, the modifications in core autophagy genes also facilitate AD incidence. An E122D ATG5 mutation weakens the combination of ATG5 and ATG12, resulting in reduced autophagy flux and decreased autophagosome formation [115]. Another gene is WDR45, which codes for the protein WIPI4. In mammalian cells, the WIPI4 proteins bridge PI3P production and LC3 lipidation to assist autophagosome maturation [116, 117]. Together, the well-defined roles in mitophagy demonstrate that defects in proteins related to mitochondrial quality control and homeostasis are intimately involved in the pathology of AD [118].

## 4. Autophagy/mitophagy therapeutics for AD

### 4. 1 Autophagy upregulation as therapy for AD

Plentiful previous studies suggest the performance of autophagic dysfunction in the pathogenesis of neurodegenerative diseases, including AD, because of aggregated proteins [125-128]. These ‘toxins’ are mainly degraded by both the ubiquitin-proteasome pathway and the autophagy pathway as substrates [129]. However, the former path cannot vigorously work as only unfolded substrates can pass through the proteasome barrel [130]. Double-membrane-bounded autophagosome besieges them into the center to start autophagy. The lysosome then fuses with the circinate autophagosome to create auto phagolysosome, in which multiple proteases degrade the protein complex. Numerous studies provide proof of the modulation of autophagy as a good therapeutic approach for neurodegenerative disease. The in vitro work examining the e?ect of autophagy upregulation on the clearance of aggregation-prone proteins caused by polyQ and polyA expansions, mutant tau, and ataxin-3 [131, 132] and mutant α-synuclein [133, 134] has suggested reductions in both of associated cell death and intracytoplasmic aggregates. Autophagy protects against both pro-apoptotic[135, 136] and pro-necrotic insults [137]. The protective e?ect from autophagy induction in cell models has successfully translated into a range of animal models. However, the experiments of Zhou *et al*. on *C. elegans* and mice lacking sgk-1 (serum/ glucocorticoid regulated kinase-1) illustrated that a high level of mPTP opening induced by elevated autophagy unexpectedly shortens their lifespan [138], implying side effects maybe occur while enhancing autophagy.

**Table 2:** Pharmacological agents potentially stimulate autophagy/mitophagy.

Agents	Study model	Effect	Refs
2,4-Dinitrophenol (DNP)	animal model	stimulate autophagy	[139]
rapamycin	AD mouse	reduce Aβ pathology	[45]
spermidine	human and yeast cells; nematodes	induce autophagy independent of SIRT1	[140]
urolithin A	C. elegans; mouse	induce mitophagy, prolong lifespan; increase muscle function	[141]
antibiotic	the mt-Keima mouse	provide a method to analyze mitophagy alterations	[81]
2-deoxyglucose	AD female mouse	stimulate ketogenesis and induce mild bioenergetic stress; enhance mitochondrial function; stimulate autophagy and clearance of Aβ	[142-149]
nicotinamide mononucleotide (NMN)	Mouse model of fatty liver disease; AD mouse	increase NAD^+^ pool	[150-154]
5-aminoimidazole-4-carboxamide ribonucleotide (AICAR)	myopathy mouse	activates AMPK to acts on PGC-1	[155-157]
actinonin	Caenorhabditis elegans	reverse memory impairment	[77]
mdivi1	excitotoxic mouse	enhance DRP1 activity	[158-164]

Numerous autophagy-modulating agents developed to treat AD, including the mechanistic target of rapamycin (mTOR)-dependent and mTOR-independent autophagy-including agents (see Table 2) [165]. The master metabolic regulator mTOR can formulate two di?erent functional complexes. The researcher believes that mTOR complex 1 (mTORC1) inhibits autophagy, while mTORC2 may differentially regulate autophagy/ mitophagy under different conditions [138, 166]. Rapamycin was the first drug to be identified as an autophagy inducer[167, 168]. Although the mTOR pathway is involved in a wide range of cellular functions, the therapeutic e?ects of rapamycin in models of neurodegenerative disease are predominantly autophagy-mediated [132, 166]. The restricted absorption of rapamycin has driven the development of many rapalogs such as everolimus (RAD001), temsirolimus (CCI-779), and ridaforolimus (AP23573). To date, it is these rapalogs that have investigated due to their potential therapeutic value in the treatment of neurodegenerative diseases. ATP-competitive mTOR inhibitors are newly developed mTOR inhibitors [169]. Stimulation of the AMPK pathway upregulates autophagy as an mTOR-dependent manner [170]. Metformin is an AMPK activator with therapeutic potential in neurodegenerative disease [171]. There is no doubt that further testing of such compounds in different AD animal models are necessary for their implementation in AD patient iPSC-derived neurons and glial cells. What is noticeable is that autophagy inducers are unlikely to be pancreatically linked for many neurodegenerative diseases [172], which, for instance, can be seen by short-term fasting [173].

### 4. 2 Enhancing mitophagy as a novel therapeutic strategy for AD

Accumulating studies suggest that dysfunctional mitochondria are mainly due to impaired mitophagy in neurons in AD [83, 174-177]. The ‘vicious cycle’ hypothesis proposed that loss-of-function mitophagy and Aβ and p-Tau, the biomarkers in AD pathophysiology, strongly influence each other [103, 114]. Moreover, the ‘vicious cycle’ experiments state that Aβ-dependent neuronal hyperactivity supports circuit dysfunction in the early stages of AD [178]. Recently, Fang and his colleagues successfully stimulated mitophagy and reversed memory impairment using NAD^+^supplementation, urolithin A and action in both Aβ and tau Caenorhabditis elegans models [77]. The stimulation of mitophagy affected the PINK1-, PDR1- or DCT1-dependent pathways. In human neurons derived from the hippocampus of AD patients and in AD animal models, enhanced mitophagy can even diminish insoluble Aβ and prevent cognitive impairment in AD mouse model through the suppression of neuroinflammation and microglial phagocytosis of Aβ plaques [77]. These findings predict that enhancing mitophagy could be a novel approach to delay or even treat AD [179, 180]. To this end, plentiful pharmacological agents have been examined in preclinical studies [181-183].

Research over the last few decades has extended understanding of nicotinamide adenine dinucleotide (NAD^+^) from a vital redox carrier and energy provider to a critical signaling molecule that is involved in the regulation of a multitude of fundamental cellular processes. To date, NAD^+^ plays vital roles in gene expression, DNA repair, calcium signaling, cell cycle regulation, mitochondrial homeostasis and neuronal maintenance and plasticity [184]. NAD^+^ is also a substrate for several families of regulatory proteins, such as poly (ADP-ribose) polymerases (PARPs), CD38/CD73, Sirtuins, and SARM1 [184, 185]. At the molecular level, the NAD^+^-dependent signaling events differ from hydride transfer since these NAD^+^-consuming enzymes cleave NAD^+^ into an ADP-ribosyl moiety and nicotinamide. Therefore, non-redox functions of NAD^+^ require continuous biosynthesis of the dinucleotide.

As an essential cellular metabolite, NAD^+^ exists in all living cells, including brain cells, where it plays fundamental roles in neuroplasticity and cellular stress resistance [186]. Because neurons consume relatively large amounts of energy, they are susceptible to decreased NAD^+^ levels as well as the impairment of ATP production [16, 85]. Furthermore, NAD^+^ affects neuronal health and survival through regulation of the balance between mitochondrial biogenesis and mitophagy, which are controlled by the NAD^+^/SIRT1-PGC-1α pathway and the DAF-16/FOXO3 pathway [85, 87]. Decreased NAD^+^ levels can compromise mitophagy and trigger the accumulation of misfolded proteins leading to neuronal death [87, 187]. Cellular NAD^+^ levels are affected by several NAD^+^-consuming enzymes which have links to AD [188].

NAD^+^ levels decline in the AD brain in a few possible ways. PARP1 is an enzyme that responds to DNA strand breaks by catalyzing poly ADP-ribosylation (PARylation) of target proteins using NAD^+^ as a cofactor [85, 188, 189]. While PARP1 was localizing the nucleus, it is now known to also localize to mitochondria in cells under stress where it can PARylate electron transport chain proteins. Levels of PARP1 activity are increased, and PARylated proteins accumulate in brain tissue samples from vulnerable brain regions of AD patients [190]. Therefore, oxidative stress is a likely trigger for PARP1 activation in AD, which may occur both upstream and downstream of Aβ accumulation. CD38 is a multifunctional enzyme that catalyzes the synthesis and hydrolysis of cyclic ADP-ribose (cADPR) from NAD^+^to ADP-ribose and mediates Ca^2+^ release from the endoplasmic reticulum. Importantly, PARP1 and CD38 activity may lead to decreased NAD^+^ levels and thus lower sirtuin activity. Treatment of neural cells with a PARP1 inhibitor can protect them against mitochondrial dysfunction and cell death caused by Aβ [191]. APP/PS1 double mutant transgenic mice that lack CD38 exhibit reduced levels of Aβ in their brains and improved learning and memory [192], consistent with NAD^+^ depletion in the promotion of amyloidogenesis in this mouse model of AD. Many researchers suggest that NAD^+^ deficiency in AD, possibly caused by PARP1 activation due to increased oxidative stress-mediated DNA damage, leads to decreased mitophagy, decreased sirtuin activity, and mitochondrial dysfunction.

### 4. 3 Other therapeutic strategies for AD

In addition to pharmacological agents, lifestyle interventions also can stimulate mitophagy to protect the mitochondrial population. It has been evaluated in animal models and even in MCI (mild cognitive impairment) or AD patients. Experiments using rodents show that fasting can reduce ROS and inflammation, optimize energy metabolism, and enhance autophagy [173, 193]. For example, fasting for two days can make GFC-LC3 transgenic mice have more autophagosomes in the neurons of the cerebral cortex. Studies of human subjects and animal models demonstrate that regular exercise provides robust beneficial effects, which in turn can reduce the risk of AD [194]. Vaynman et al. showed that by upregulating mitochondrial uncoupling protein 2 (UCP2) levels in the hippocampus, exercise could lower ROS and active autophagy. Caloric restriction (CR) also can enhance mitochondrial function. On the one hand, CR can decrease ROS and reduce oxidative DNA damage; on the other hand, CR can induce the expression of many sirtuins, especially SIRT1. Its overexpression can extend the life span and improve the syndromes of AD [195]. In sum, the enhanced mitophagy can reclaim the dysfunctional mitochondria to maintain high-quality mitochondrial homeostasis in neurons.

## 5. Conclusions

In this review, we have delineated mitochondrial biology, the average and defective autophagy and mitophagy in AD, highlighting their pathogenesis and corresponding therapeutic strategies. In AD, mitochondrial dysfunction and the bioenergetic deficit contribute to the Aβ and p-Tau pathologies; in turn, these two pathologies promote mitochondrial defects. As a consequence, a fundamental characteristic of AD is the impairment of mitochondria, which has been ascertained in both sporadic and familiar types of samples as well as in animal models. As stated above, pharmacological agents, fasting, physical exercise, and caloric restriction can reverse this impairment. The main target of these methods is to enhance autophagy and mitophagy. Mitophagy plays a fundamental role in mitochondrial quality control and homeostasis, and the pathological consequences of its misregulation demonstrate its importance. However, the exact positions of mitophagy in AD etiology are still unclear as multiple steps are affected. Cells regulate mitochondrial degradation not only through control of the mitophagy machinery but also through delicate tuning of mitochondrial fusion and fission [34, 76]. It remains to see whether other cellular processes linked with mitochondria also have a role to play in mitophagy regulation. Further understanding of the mechanisms involved in mitochondrial quality control will hopefully hold therapeutic potential for AD.

Here we would like to recommend NAD^+^ to implement further research in drug discovery for AD. The importance of the regulatory roles of NAD^+^ has established in many excellent studies. While impressive progress has made regarding the mechanisms of NAD^+^-dependent inhibition of AD pathology and the restoration of cognitive function, some critical questions remain unanswered. For example, cellular NAD^+^ biosynthesis and consumption compartmentalized in the cell, but how these organellar NAD^+^ pools are established and maintained in neurons and glial cells, including astrocytes and microglia, remains unknown. Furthermore, what are the differences between different NAD^+^ precursors, NMN, NR, nicotinic acid, and nicotinamide, in treating AD? The pharmacokinetics and potential toxicity data of NAD^+^augmentation strategies in AD patients need to obtain in the clinic.AD might be not only a brain disorder but also a systemic disease with widespread abnormalities beyond the brain. Thus, systemic factors might interact with brain-related elements to modify the AD process. AD diagnosis and treatment should have a corresponding focus not only on pathological changes in the brain but also on peripheral abnormalities, which vary among individuals. Identifying these peripheral abnormalities might offer new opportunities for diagnosis of early AD and lead to the design of specific treatment strategies for individuals with AD at different stages.

## 6. Future perspectives

In the past 20 years, most of the drugs tested in the clinic for AD have targeted the Aβ accumulation; however, none of these anti-Aβ therapies overcome the central problem [196]. Today, a promising alternative option for AD therapeutics is to maintain mitochondrial homeostasis by enhancing autophagy and stimulating mitophagy. Dysfunctional mitophagy can increase Aβ and Tau pathologies, while aggregating Aβ can impair neuronal mitophagy in reverse. These outcomes indicate pivotal roles for mitophagy dysfunction, both upstream and downstream of Aβ and Tau pathways [16]. In all, different theories such as “amyloid plaques, NFT, mitochondrial dysfunction, neuroinflammation, comprised autophagy et al.” result in AD etiologies that interact with each other [197]. The ‘Chicken and egg’ relationships between different hallmarks of AD need to establish, and specific therapy should direct to target the reason for the neuronal insult and not the host response. Meanwhile, we can seek some clues from the treatment of other diseases like cancers, diabetes and rheumatoid arthritis.
